# Diagnosis, monitoring, and management of axial spondyloarthritis

**DOI:** 10.1007/s00296-024-05615-3

**Published:** 2024-05-17

**Authors:** Olena Zimba, Burhan Fatih Kocyigit, Mariusz Korkosz

**Affiliations:** 1 Department of Rheumatology, Immunology and Internal Medicine, University Hospital in Krakow, Krakow, Poland; 2 National Institute of Geriatrics, Rheumatology and Rehabilitation, Warsaw, Poland; 3 Department of Internal Medicine N2, Danylo Halytsky Lviv National Medical University, Lviv, Ukraine; 4 Department of Physical Medicine and Rehabilitation, University of Health Sciences, Adana City Research and Training Hospital, Adana, Türkiye; 5 Department of Rheumatology and Immunology, Jagiellonian University Medical College, Jakubowskiego 2 Str., 30-688, Kraków, Poland

**Keywords:** Axial spondyloarthritis, Spondylarthritis, Ankylosing spondylitis, Diagnosis, Disease management, Treatment, Telehealth

## Abstract

Axial spondyloarthritis (axSpA) is a chronic condition predominantly affecting the spine and sacroiliac joints. This article provides an in-depth overview of the current approaches to diagnosing, monitoring, and managing axSpA, including insights into developing terminology and diagnostic difficulties. A substantial portion of the debate focuses on the challenging diagnostic procedure, noting the difficulty of detecting axSpA early, particularly before the appearance of radiologic structural changes. Despite normal laboratory parameters, more than half of axSpA patients experience symptoms. X-ray and magnetic resonance imaging (MRI) are essential for evaluating structural damage and inflammation. MRI can be beneficial when there is no visible structural damage on X-ray as it can help unravel bone marrow edema (BME) as a sign of ongoing inflammation. The management covers both non-pharmacological and pharmacological approaches. Lifestyle modifications, physical activity, and patient education are essential components of the management. Pharmacological therapy, including nonsteroidal anti-inflammatory drugs (NSAIDs) and biologic disease-modifying anti-rheumatic drugs (bDMARDs), are explored, emphasizing individualized treatment. To effectively manage axSpA, a comprehensive and well-coordinated approach is necessary, emphasizing the significance of a multidisciplinary team. Telehealth applications play a growing role in axSpA management, notably in reducing diagnostic delays and facilitating remote monitoring. In conclusion, this article underlines diagnostic complexities and emphasizes the changing strategy of axSpA treatment. The nuanced understanding offered here is designed to guide clinicians, researchers, and healthcare providers toward a more comprehensive approach to axSpA diagnosis and care.

## Introduction

Spondyloarthritis (SpA) is a long-lasting condition, delineated into two discernible manifestations: axial SpA (axSpA), predominantly affecting the spine and sacroiliac joints, and peripheral SpA (pSpA), exerting its influence on the peripheral joints and related structures. The paramount manifestation of axSpA is radiographic axSpA (r-axSpA), synonymous with ankylosing spondylitis (AS), traditionally identified using the modified New York criteria [1]. The distinctive feature of r-axSpA is  radiographic sacroiliitis. Nevertheless, the phrase is inaccurately coined as sacroiliitis, specifically described as the inflammation of the sacroiliac joint. Radiographs solely identify structural damage rather than inflammation [2, 3].

The main focus has been on the existing challenges in identifying and classifying patients with axial symptoms of SpA who do not meet the sacroiliitis criteria [4]. Patients without structural damage were first categorized as having early or preradiographic axSpA. Later on, the wording was modified to non-radiographic axial spondyloarthritis (nr-axSpA) because not all patients develop AS, what is now referred to as r-axSpA [5].

The Assessment in SpondyloArthritis International Society (ASAS) introduced the term 'axial spondyloarthritis' to encompass the disease's complete range, including patients with evident radiographic damage and those without [6].

While some studies suggest a SpA prevalence below 1% [7–9], it is thought that certain geographical regions can have a prevalence as high as 1.4% [10]. The discrepancies can be attributed to variations in the methodology and sample size, yet, it is widely recognized that the prevalence is significantly influenced by the underlying prevalence of the human leukocyte antigen (HLA)-B27 [11]. The sex balance of SpA varies, with a male-to-female ratio of 2–3:1 [12]. There is a more even distribution of sexes in nr-axSpA, and the proportion of nr-axSpA is progressively rising, possibly due to improved diagnosis [13].

## Aim

The aim of the article is to provide a comprehensive review of current understanding, obstacles, and advancements in the diagnosis, classification, monitoring, management, and involvement of multidisciplinary teams, and the incorporation of telehealth into the care of patients with axSpA. Through an in-depth exploration of the literature and research findings, this article seeks to elucidate the complexities of axSpA diagnosis, emphasize the importance of proper classification criteria, discuss various methods of monitoring disease activity and progression, assess both non-pharmacological and pharmacological treatment approaches, and highlight the crucial function of multidisciplinary teams.

## Search strategy

Relevant articles were retrieved from Medline/PubMed, Scopus, and Web of Science using the search terms "spondyloarthritis" or "ankylosing spondylitis", and "diagnosis" or "classification" or "criteria" or "guidelines" or "imaging" or "treatment" or "management" or "telehealth". Only articles published in English until January 2024 were considered. No specific time frame was established. Following the listing of the articles, those that were not directly relevant to the issue were excluded. After the exclusion process, the authors analyzed the articles they deemed appropriate.

## Diagnosis

AxSpA presents with a diverse range of clinical manifestations. Regrettably, no characteristic derived from the medical records, physical exam, test results, or radiologic evaluations possesses the necessary accuracy to diagnose axSpA definitively. Diagnosis entails identifying a collection of characteristic patterns and qualities that, when considered collectively, offer enough evidence to confirm the presence of axSpA [14].

The physician typically evaluates the probability of axSpA by balancing positive and negative test results in the accurate diagnostic procedure [15]. Early-stage identification of axSpA can provide challenges, especially before the conclusive diagnosis of radiological sacroiliitis. There is a suggestion to diagnose individuals at early phases using probability estimates derived from a 5% pretest probability in individuals with chronic back pain [16, 17].

Due to the complexities of mathematical modeling, Rudwaleit et al. [4] focused on utilizing positive likelihood ratios. The parameters exhibiting the most heightened positive likelihood ratio values include sacroiliitis verified through X-ray or magnetic resonance imaging (MRI), family history or HLA-B27 positivity, and acute anterior uveitis [4]. However, absence of a specific characteristic can occasionally help rule out a diagnosis in routine diagnostic processes. In the case of axSpA, the non-existence of certain parameters significantly diminishes the likelihood of the diagnosis. These involve the absence of HLA-B27, negative MRI result, mismatch to the character of inflammatory back pain, typical acute phase reactants, unresponsiveness to NSAIDs, and, presumably, negative family history. Moreover, certain clinical signs may be absent in initial assessments. The clinical picture during the progression of the disease is primarily associated with the duration of the disease. These signs encompass inflammation in peripheral joints and soft tissues, ophthalmological involvement, skin and intestinal manifestations. The inclusion of these parameters contributes to a more accurate diagnosis. However, if they are not detected during the initial stages of the disease, they can be neglected [18].

Laboratory parameters are an integral part of diagnosing axSpA by assisting in thoroughly evaluating inflammatory processes and adding to the entire diagnostic framework. Assessment of acute-phase reactants is a standard practice [19, 20]. Nevertheless, a significant proportion of individuals with axSpA experience symptoms even when their acute-phase reactants are within normal range, with estimates suggesting that this occurs in up to 60% of patients [21]. Furthermore, the existence of HLA-B27, while not limited to axSpA, is an important indicator linked to axSpA. Although not used as a single diagnostic tool, the presence of HLA-B27 antigen is consistent with the diagnostic algorithm's clinical signs and imaging results [22, 23]. Incorporating these laboratory markers into a thorough clinical assessment is crucial for prompt diagnosis of axSpA.

Using radiologic assessment is essential for accurately and promptly diagnosing and differentiating axSpA. Since the disease commonly damages the sacroiliac joints, imaging these structures is essential in diagnosing [24].

For initial evaluation of suspected axSpA, it is advisable to arrange sacroiliac joints X-ray [25]. This is owing to the fact that X-rays are readily accessible. Conversely, the sensitivity of this test is limited, particularly in individuals who have experienced symptoms for a short period [26]. A significant concern associated with this approach is the limited reliability due to the intricate structure and individual variations in the visual representation of sacroiliac joints on conventional X-rays. The substantial inter-observer variability is also one of the drawback in the assessment of SIJ X-ray [27].

Computerized tomography (CT) is an emerging radiologic method widely recognized as the most reliable way to identify structural damage [28]. However, conventional CT cannot evaluate alterations related to inflammation in the sacroiliac joint [29].

If conventional radiographs fail to detect any signs of sacroilitis, and there is still suspicion of axSpA, it is recommended to perform sacroiliac joint MRI [25, 30]. The decision to incorporate the spine in the MRI depends on the clinical manifestations and potential diagnoses. The addition of spinal MRI does not significantly contribute to the diagnosis compared to sacroiliac joint MRI [31]**.** An essential benefit of MRI is its ability to identify active inflammatory changes, namely BME that might emerge before any structural damage. Furthermore, MRI is highly effective in accurately detecting structural alterations. Identifying BME of the sacroiliac joint enhances the probability of diagnosing axSpA, particularly when accompanied by structural alterations [32, 33]. Nevertheless, it is crucial to acknowledge that BME is not as indicative of axSpA as previously believed. Various disorders can lead to BME, e.g. intensive physical training  and mechanical back pain [34].

## Classification criteria

Classification criteria for axSpA have been developed for research purposes. In order to facilitate research, classification criteria are set consistently with the primary goal of producing clearly defined and homogeneous groups. The criteria used for classifying axSpA were established by ASAS in 2009 [35]. The ASAS axSpA criteria include sacroiliac joint MRI and apply to the full range of the condition (nr-axSpA and r-axSpA) [36]. An axSpA patient must exhibit persistent back pain lasting more than three months before the age of 45 in order to fulfill the entrance condition. Furthermore, to meet the criteria, a patient must exhibit sacroiliitis on imaging along with at least one additional SpA characteristic. Alternatively, a patient must test positive for HLA-B27 and have at least two SpA features.

Classification criteria may appear simple to apply as diagnostic criteria. Nevertheless, their utilization for this objective is not advisable. Employing classification criteria for the diagnostic process not only disregards the crucial matter of differential diagnosis but also results in an unacceptably high rate of misdiagnoses, including both axSpA patients who are inaccurately overlooked and patients without axSpA who are inaccurately labeled as axSpA. The inherent restrictions in sensitivity and specificity are partially due to the categorical nature of these criteria that only determine if they are met [37, 38]. Additionally, several scholars contend that all characteristics are equally important, regardless of their varying value. The primary rationale for assigning equal weight was to ensure simplification and prioritize the ease of execution [39].

## Monitoring

A wide range of options are currently accessible for monitoring axSpA. Most of the methods employed in axSpA rely on laboratory investigations, radiologic evaluations, and patient-reported results [40, 41].

Only patient-reported metrics are used in the Bath Ankylosing Spondylitis Disease Activity Index (BASDAI) [42] to gauge disease activity in SpA. It was described in 1994 and has been utilized frequently, but certain restrictions exist. It does not consider healthcare professionals' opinion on the condition or the influence of individual clinical variables. The BASDAI is not sensitive enough to detect actual inflammation. The BASDAI has benefits, including its user-friendly nature and broad acceptance.

The Ankylosing Spondylitis Disease Activity Score (ASDAS) was developed as a composite index. This tool is characterized by integrating patient reports and acute phase reactants [43, 44]. The ASAS encourages the implementation of ASDAS-CRP in clinical settings and research activities. However, ESR-version may also be utilized. ASAS has established confirmed cut-off degrees for disease activity assertions. [45]. A decline of at least 1.1 across two evaluations is deemed a clinically important improvement, while a reduction of 2.0 is classified as a major improvement. Disease flare is characterized by a rise in the ASDAS of 0.9 or higher, in contrast to the prior evaluation [46]. Additional criteria for assessing response and remission include ASAS20, ASAS40, ASAS 5/6, ASAS partial remission, and BASDAI 50 response [47–49].

The evaluation of an individual's physical function is a prerequisite for axSpA. During this process, the Bath Ankylosing Spondylitis Functional Index (BASFI) is the instrument that is utilized rather frequently. BASFI comprises ten questions, eight of which pertain to various parts of functional anatomy and two of which pertain to the capacity to deal with day-to-day life. Each of the ten questions contributes to the overall BASFI score (by averaging), which can vary from 0 to 10 [50].

One of the severe consequences of axSpA is the deterioration of spinal mobility, and it is recommended that this aspect be incorporated into the evaluation methods carried out on patients in clinical practices [24, 51]. Bath Ankylosing Spondylitis Metrology Index (BASMI) is a commonly used clinical tool for assessing spinal mobility. It includes examinations of movement in the spine and hips [52]. BASMI is a quantitative assessment tool used to quantify the course of spinal progression, explicitly focusing on non-imaging aspects. While BASMI measurements may be relatively straightforward to conduct, they require a significant amount of time and should be done in person by qualified healthcare professionals [53].

SpA-specific measures do not sufficiently evaluate the comprehensive assessment of disabilities, boundaries, and limitations in performing daily tasks or social engagement. The ASAS Health Index was created to evaluate complex well-being and quality of life in axSpA patients [54]. The ASAS Health Index is derived from 251 elements gathered via surveys on axSpA. It comprises 17 specific points [55, 56]. The main advantages and disadvantages of monitoring tools are summarized in Table 1.

**Table 1:** The main advantages and disadvantages of monitoring tools

Tool	Advantages	Disadvantages
BASDAI	• BASDAI has a user-friendly structure. • BASDAI is widely accepted. • BASDAI allows for a quick evaluation of disease activity.	• BASDAI does not consider healthcare professionals' opinion. • BASDAI is not sensitive enough to detect actual inflammation. • BASDAI lacks objective measures. • BASDAI is subjective.
ASDAS	• ASDAS is a composite index that merges patient-reported outcomes with acute-phase reactants. • ASDAS utilizes a standardized scoring structure. • ASDAS offers a quantitative and objective measurement of disease activity.	• The complexity may be problematic in regular clinical practice. • ASDAS is dependent on laboratory tests.
BASFI	• BASFI is an easily applicable tool. • It is a globally accepted and valid tool. • BASFI shows sensitivity to changes in functional status over time.	• BASFI is based on subjective self-assessment. • BASFI necessitates individuals to recall.
BASMI	• BASMI provides an objective assessment of spinal mobility. • BASMI uses standardized protocol. • BASMI evaluates multiple anatomical locations.	• The assessment is based solely on clinical examination, without the utilization of imaging tools. • BASMI depends on the patient's ability to cooperate. • BASMI may encounter difficulty in detecting minor changes.
ASAS Health Index	• ASAS Health Index provides a comprehensive assessment of health and well-being. • Aside from physical symptoms, the ASAS Health Index covers psychological and social impacts. • ASAS Health Index adheres to the ideals of patient-centered care.	• ASAS Health Index is based on subjective responses. • It requires patients to recall. • It may lack the objectivity.

*BASDAI* Bath Ankylosing Spondylitis Disease Activity Index, *ASDAS* Ankylosing Spondylitis Disease Activity Score, *BASFI* Bath Ankylosing Spondylitis Functional Index, *BASMI* Bath Ankylosing Spondylitis Metrology Index, *ASAS* Assessment in SpondyloArthritis International Society

Enthesitis is a usual feature of axSpA. The initial method for evaluating enthesitis is referred to as the Mander Enthesitis Index (MEI) [57]. The MEI quantifies the patient's reaction following localized exertion at 66 enthesal locations. The scoring is done. As an alternative, there have been suggestions for using more concise indices covering the Berlin Enthesitis Index (BEI) [58], Spondyloarthritis Research Consortium of Canada [59], Leeds Index [60], and the Maastricht AS Enthesitis Score (MASES) [61].

Only 30–40% of those affected exhibit increased acute-phase reactants, and it is essential to note that a normal value does not necessarily mean that inflammation is absent. When axSpA is accompanied with peripheral joint inflammation or inflammatory bowel disease, there is a higher occurrence of increased acute-phase reactants [21].

As previously stated, MRI is a highly sensitive tool for identifying signs of inflammation in the sacroiliac joints and spine. Nevertheless, regularly using these methods in rheumatology practice to monitor axSpA is not advisable as their incremental benefit compared to more suitable instruments has not been definitively established [25]. Various scoring systems have been created for scientific investigation. These scores are commonly employed to evaluate the treatment efficacy in clinical trials [62, 63].

The typical method for evaluating structural damage is through sacroiliac joint and spine radiography. However, there is no consensus on the specific procedure for conducting this assessment in clinical settings [25]. Different scoring methodologies have been suggested to evaluate the extent of radiological damage in axSpA. The most reliable and widely acknowledged tool is the modified Stoke AS Spine Score (mSASSS) [64, 65]. The mSASSS utilizes defined criteria to grade cervical and lumbar spine lateral plain radiographs. The overall score spans from 0 to 72.

## Management

The care of axSpA patients involves both non-pharmacological and pharmacological procedures. An individualized strategy, tailored to particular requirements and backed by scientific evidence, is essential [66]. Integrating both intervention groups (non-pharmacological and pharmacological) is crucial for axSpA [67]. The treatment strategy should strive to attain the utmost level of quality of life and patients’ health status assessment [68]. Due to the inflammatory nature of axSpA, a considerable amount of existing treatments focus on decreasing the inflammatory load. Furthermore, considering the influence of disease severity on both structural damage and functional status, it is crucial to prioritize the handling of inflammation in the treatment [69, 70]. A pre-established, targeted therapeutic approach, mutually agreed upon by the patient and physician, proves beneficial. In axSpA, the link between increasing ASDAS scores and a higher incidence of syndesmophytes indicates that ASDAS is a suitable focus for intervention [71].

### Non-pharmacological therapies

AxSpA patients should engage in a comprehensive educational initiative regarding the disease. The main goal of self-management is to engage and enable patients to collaborate proactively. In a broader sense, patient education should encompass knowledge regarding the disorder, its signs and identification, progression, available treatment alternatives, and future directions [72].

Physical activity—exercise is a fundamental aspect of treating axSpA. Although exercise is typically included in the management approach, qualitative research indicates compliance improves when supervised [67, 73]. Although exercise interventions have shown impressive outcomes in investigations, aggressive exercise programs may adversely affect axSpA patients. Mechanical overloading can potentially increase inflammation and the development of additional bone formation in the enthesal and joint areas [73]. Studies utilizing computer modeling have demonstrated a model of syndesmophyte formation in areas of high mechanical stress in the spine in patients with long-term AS using computerized tomography scanning [74]. However, a definitive link between exercise and the development of syndesmophytes has not been established. Hence, exercise, a fundamental component of axSpA management, should not be abandoned.

Modifying detrimental lifestyle behaviors is crucial in the management of axSpA. Suggestions for lifestyle practices to prevent disease progression encompass adhering to a nutritious and well-balanced diet, keeping an optimal body weight [75]. Research has demonstrated that smoking is a contributing factor to the advancement of spinal inflammation and structural damage in axSpA [76, 77]. Hence, it is essential to motivate axSpA patients to quit smoking.

### Pharmacological therapies

NSAIDs are the primary choice for managing axSpA with a pharmacologic approach. Individuals who suffer from pain and stiffness should carefully evaluate the hazards and benefits of utilizing NSAIDs at the maximum acceptable and tolerable doses [66]. If it is deemed necessary to control signs, the ongoing utilization of NSAIDs can be contemplated in individuals who suffer from symptoms. However, around one-third of patients demonstrate either nonresponsiveness or intolerance toward NSAIDs [78]. Concerning the effectiveness of NSAIDs in reducing structural damage, research up to this point has produced contradictory results. Compared to patients who received NSAIDs as needed, those with r-axSpA who were treated continually for two years with NSAIDs (primarily celecoxib) had retarded radiological progress [79]. This finding was not supported in a subsequent investigation of diclofenac [80]. Furthermore, continuous celecoxib and golimumab in combination therapy did not provide any meaningful advantage over golimumab alone in reducing structural deterioration in r-axSpA over two years [81]. Clinical improvement resulting from the administration of the full dose of NSAIDs is frequently noted within two weeks. If there is no sufficient response within this time, it is advisable to consider using a second NSAID. Currently, there is inadequate data to determine if alternating between standard NSAIDs and COX-2 inhibitors is superior to using a second NSAID from the same category [82].

In patients who continue to experience high disease activity after taking two NSAIDs at adequate doses and duration, biologic disease-modifying antirheumatic drugs (bDMARDs) or targeted synthetic disease-modifying antirheumatic drugs (tsDMARDs) can be considered [66]. Two classes of bDMARDs, tumor necrosis factor inhibitor (TNFi) and interleukin 17 inhibitor (IL-17i), and one class of tsDMARD, janus kinase inhibitor (JAKi), have been approved. Without direct comparative clinical trials, it is challenging to determine the relative effectiveness of different choices and establish a clear priority. Nevertheless, it is widely recognized that initiating treatment with a TNFi or IL-17i is a customary approach [66]. This assertion is supported by extensive prior experience with TNFi and IL-17i, robust and comprehensive evidence, a wealth of safety data, and considerable familiarity with these medications in patients with numerous comorbidities, typically excluded to ensure homogeneity in high-quality trials.

TNFi has been a treatment option, and infliximab, etanercept, adalimumab, golimumab, and certolizumab pegol have been approved for axSpA. Infliximab has been approved exclusively for r-axSpA whereas the others have been approved for nr-axSpA and r-axSpA [83]. Evidence indicates this class of drugs has substantial benefits over placebo in controlling disease activity, enhancing functionality, and reaching partial remission [84]. According to an extensive European database of individuals with axSpA who started their initial TNFi medication as part of their regular management, 27% of individuals reached ASDAS inactive disease following six months, and 59% obtained BASDAI < 4. After a year, four out of five individuals were still receiving medication [85]. The efficacy of TNFi drugs has also been evidenced by a decrease in inflammation observed on sacroiliac MRI [86]. Comparable patterns have been exhibited in reducing inflammation in the spinal vertebrae when subjected to TNFi therapy [87].

Incorporating IL-17i in rheumatology has expanded the pharmacological armamentarium available for axSpA. Currently, secukinumab and ixekizumab have been approved [66]. Secukinumab and ixekizumab were more effective than placebo in an evaluation based on ASAS40 responses [88]. IL-17i has demonstrated effectiveness in individuals who previously received TNFi treatment, albeit with reduced efficacy compared to those without TNFi treatment [89]. Although TNFi and IL-17i alleviate axSpA symptoms with favorable safety characteristics, there is currently no sufficient data to suggest that one is more effective.

Tofacitinib exhibited superior efficacy over placebo in active axSpA at week 16 as determined by an analysis based on the ASAS40 response [90]. The investigation on upadacitinib's effects on active r-axSpA yielded comparable outcomes [91]. An advantage of these treatment options is their oral administration. Moreover, upadacitinib demonstrated a substantial enhancement in nr-axSpA symptoms as compared to placebo during the week 14 evaluation [92].

**Fig. 1:**
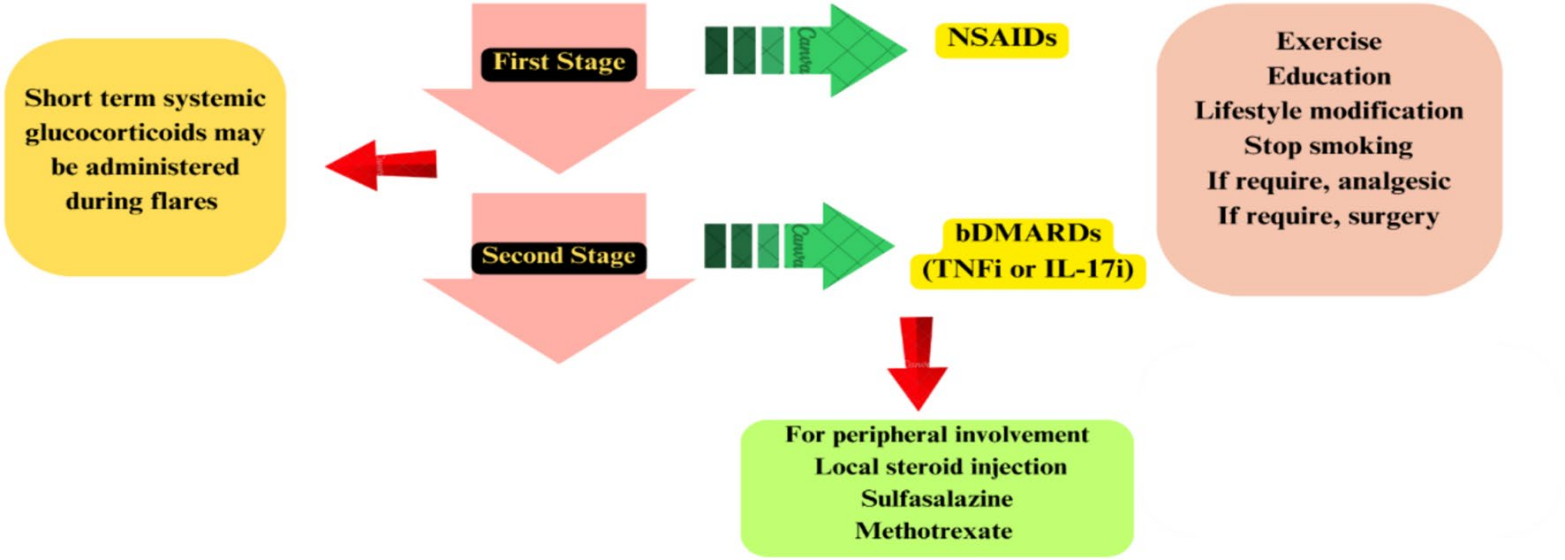
Axial spondyloarthritis treatment approach. *NSAIDs* non-steroidal anti-inflammatory drugs, *bDMARDs* biolgical disease-modifying anti-rheumatic drugs, *TNFi* tumour necrosis factor inhibitors; *IL-17i* IL-17 inhibitors

There is insufficient evidence to support the effectiveness of csDMARDs in managing axSpA. Extended care for axial signs is not supportive of systemic glucocorticoids. However, systemic glucocorticoids may be administered during disease flares marked by increased disease activity, inflammation, and pain. There is evidence to suggest the efficacy of high dosages of systemic glucorticoids, and this could be a beneficial addition to the axial SpA armamentarium [93]. In some cases, this method may be effective for short-term disease control. Short-term use of high-dose or pulsed systemic glucocorticoids may be part of SpA's therapeutic repertory to handle severe axSpA flares that are unresponsive and debilitating to NSAIDs when biologics are unavailable or inappropriate. When administered in the short term, its benefits in reducing severe pain and stiffness and improving mobility are expected to outweigh bone loss and other adverse effects [94]. Additionally, intra-articular injections may be taken into consideration. In certain instances, local injections can be efficacious for patients experiencing enthesitis. Paracetamol and opioids can be utilized for pain that continues despite the standard treatment strategy [66] (Fig. 1).

## Optimal treatment options for particular subsets

Although bDMARDs and tsDMARDs have comparable effectiveness in managing the axSpA, there are notable distinctions in their effectiveness for non-musculoskeletal manifestations [95]. In cases of recurring uveitis or persistent inflammatory bowel disease, anti-TNF monoclonal antibodies are preferable [96]. Although etanercept has presented contradictory outcomes, monoclonal TNFi (infliximab, adalimumab, and golimumab) treatment can be evaluated for individuals who do not benefit from conventional treatment in managing acute anterior uveitis [66]. When analyzing etanercept and secukinumab for uveitis exacerbation prevention, registry data investigations demonstrated that monoclonal anti-TNF agents were superior [97]. IL-17i is not recommended in clinical scenarios where axSpA is coupled with inflammatory bowel disease [98]. Considering the positive impact of IL-17i on skin signs of psoriasis, they might be a more suitable option than TNF inhibitors for individuals with severe skin conditions [99, 100]. Evidence has demonstrated JAKi's tofacitinib efficacy in treating chronic plaque psoriasis and inflammatory bowel disease.

## Role of the multidisciplinary team in the management

**Fig. 2:**
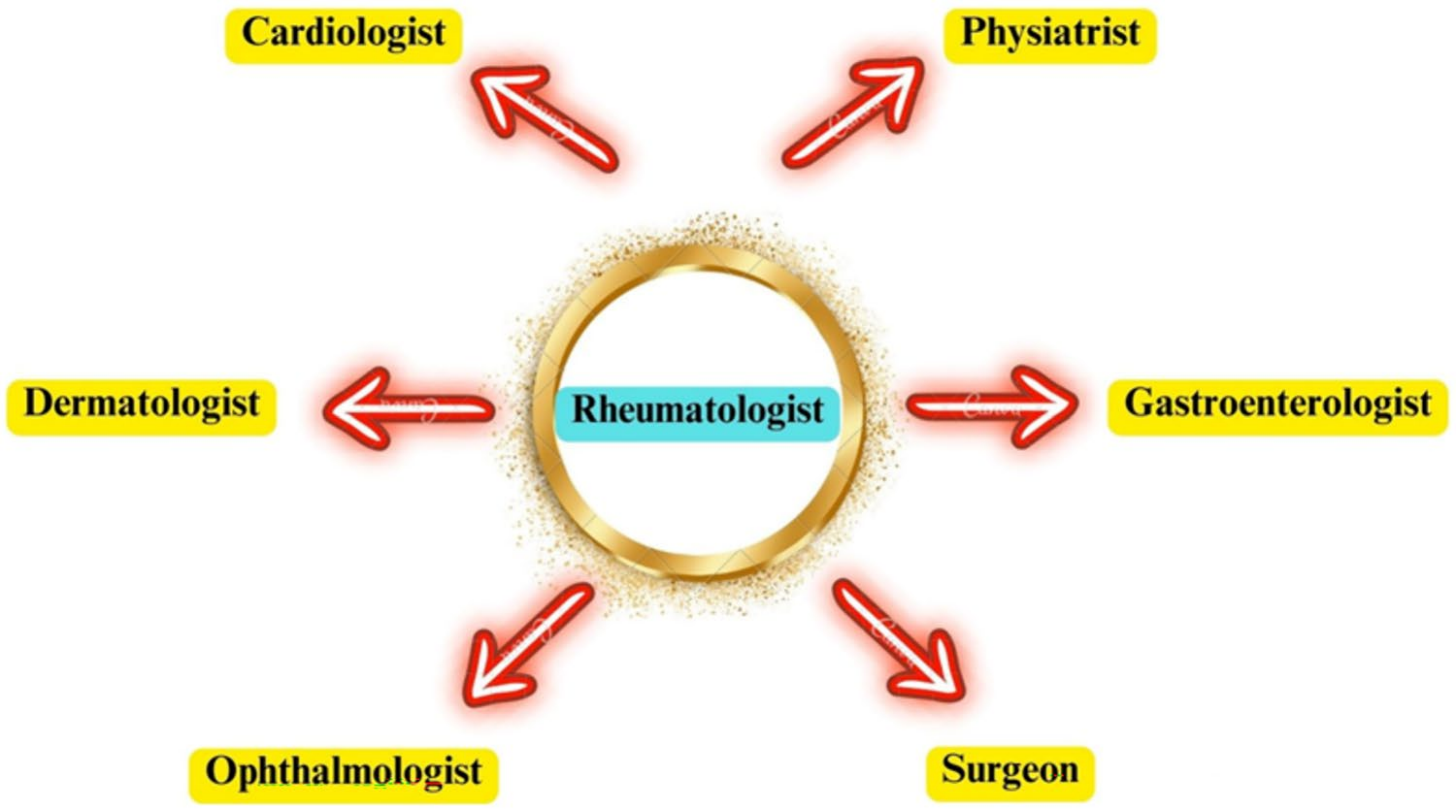
Multidisciplinary team in axial spondyloarthritis management

Effective management of axSpA requires a thorough and well-coordinated strategy, highlighting the crucial importance of a multidisciplinary team. The intricate nature of SpA, with its wide range of clinical presentations and influence on multiple facets of patients' well-being, necessitates the involvement of professionals from various healthcare disciplines to ensure efficient treatment [66, 101]. The multidisciplinary team typically comprises rheumatologists, rehabilitation specialists, physiotherapists, occupational therapists, dermatologists, gastroenterologists, ophthalmologists, cardiologists, and, if needed, orthopedic surgeons and pain medicine experts [102–104] (Fig. 2).

The primary advantage of utilizing a multidisciplinary team to manage axSpA is the capacity to effectively address each aspect of the disease. Rheumatologists are the primary professionals responsible for diagnosing, managing, and monitoring disorders [66]. Rehabilitation specialists, physiotherapists, and occupational therapists are crucial in improving physical function and mobility as part of the complete treatment strategy [105, 106]. Considering the involvement of SpA, it is evident that the input of dermatologists, gastroenterologists, ophthalmologists, and cardiologists is required. Orthopedic surgeons are typically involved in cases that require surgical procedures [107].

The effects of axSpA go beyond physical manifestations and encompass psychological and social aspects [108, 109]. Psychologists and social workers are essential members of the multidisciplinary team, as they address mental health issues and assist patients in managing the difficulties associated with living with a persistent inflammatory disease.

Continuous and coordinated interaction among team members provides an efficient and patient-focused strategy. This collaborative endeavor allows a more nuanced comprehension of the patient's condition and promotes tailored care regimens. The advantages of employing a multidisciplinary approach in managing SpA include efficient disease management, improved quality of life, and decreased long-term disability [110]. In addition, the multidisciplinary team can aid in the early identification of complications and rapid care, thereby preventing irreversible damage.

## Telehealth

Remote healthcare utilizes technological advances known as 'telehealth' operations [111]. Interaction with patients and caregivers is employed throughout the patient journey, encompassing disease assessment and monitoring various elements of the disease, such as disease severity and progression, deterioration, quality of life, and compliance [112]. Transmission can occur either synchronously, with both the health professional and patient accessible simultaneously, or asynchronously, through videos, storage-transmission of medical events, and monitoring of the patients remotely [113].

Diagnostic delay is a severe challenge in axSpA. Utilizing asynchronous telemedicine solutions can effectively address this issue. Obtaining radiologic images is crucial at this juncture [114]. Integrating electronic patient-reported outcomes allows for remote and standardized evaluation of treatment effectiveness and rapid modifications to the treatment plan. Patients with high disease activity demonstrate a strong commitment to monitoring their electronic patient-reported outcomes. Additionally, there is an agreement between the printed and digital BASDAI [115, 116]. A systematic review of rheumatic diseases concluded that remote care yielded comparable or superior results in effectiveness, safety, patient compliance, and user satisfaction outcomes compared to in-person care. However, the existing studies exhibit heterogeneity in methodology, and there is an elevated risk of bias in favor of specific outcomes [117]. In telehealth applications, online physiotherapy can be considered a worthwhile option for axSpA patients [118].

## Conclusion and future perspectives

The care of axSpA requires a comprehensive and multifaceted strategy involving diagnosis, monitoring, and therapeutic interventions. Due to the lack of a single reliable test, diagnosing axSpA remains challenging. Clinical, laboratory, and imaging aspects all contribute to the diagnosis. Monitoring axSpA demands the utilization of several assessment tools, and the involvement of a multidisciplinary team is essential in managing diverse manifestations of axSpA.

Additional endeavors should be undertaken to ascertain the axSpA's initial stages, especially where there is a risk of over-diagnosis nr-axSpA. Considering the diagnostic delay, often due to under-diagnosis, it is crucial to prioritize early detection and create appropriate interventions accordingly. To achieve early diagnosis, it is essential to strive for optimal imaging use and additional biomarkers. The most effective ways to monitor disease activity and clinical changes in conjunction with technological advancements should be identified. Attempts should also be made to clarify how to select the best patients who would benefit the most from different treatment methods.

## Data Availability

There is no stored data set associated with the article.
